# Efficacy of Obcordata A from *Aspidopterys obcordata* on Kidney Stones by Inhibiting NOX4 Expression

**DOI:** 10.3390/molecules24101957

**Published:** 2019-05-21

**Authors:** Yihang Li, Guoxu Ma, Yana Lv, Jing Su, Guang Li, Xi Chen

**Affiliations:** 1 Yunnan Branch, Institute of Medicinal Plant, Chinese Academy of Medical Sciences, Peking Union Medical College, Jinghong 666100, China; yhli@implad.ac.cn (Y.L.); ynlv@implad.ac.cn (Y.L.); jsu@implad.ac.cn (J.S.); 2 Key Laboratory of Dai and Southern Medicine of Xishuangbanna Dai Autonomous Prefecture, Jinghong 666100, China; 3 Institute of Medicinal Plant Development, Chinese Academy of Medical Sciences & Peking Union Medical College, No. 151, Malianwa North Road, Haidian District, Beijing 100193, China; gxma@implad.ac.cn

**Keywords:** *Aspidopterys obcordata*, Obcordata A, NOX4, antiurolithiatic

## Abstract

Obcordata A (OA) is a polyoxypregnane glycoside derived from the Dai medicine *Aspidopterys obcordata* vines. This study aims to investigate the efficacy of OA on renal tubular epithelial cells exposed to calcium oxalate crystals. We incubated renal tubular cells with 28 μg·cm^2^ calcium oxalate crystals for 24 h with and without OA, GKT137831, phorbol-12-myristate-13-acetate (PMA), and tocopherol. The MTT [3-(4, 5-dimethylthiazol-2-yl)-2,5-diphenyltetrazolium bromide] assay, microscopic examination, flow cytometry, and immunofluorescence staining revealed that calcium oxalate crystals decreased cell viability and elevated reactive oxygen species (ROS) levels. OA, GKT137831, and tocopherol protected cells and decreased ROS levels. However, OA did not exhibit direct DPPH scavenging ability. In addition, immunoblotting illustrated that OA inhibited the NOX4 (nicotinamide adenine dinucleotide phosphate oxidases 4) expression and downregulated the protein expression in the NOX4/ROS/p38 MAPK (p38 mitogen-activated protein kinase) pathway. The findings suggest that the cytoprotective and antioxidant effects of OA can be blocked by the NOX4 agonist PMA. In conclusion, OA could be used as a NOX4 inhibitor to prevent kidney stones.

## 1. Introduction

Nephrolithiasis, which is caused by the pathological mineralization of crystals and organic substrate in the kidneys [1,2], is a common urological disease worldwide with a lifetime incidence of 10–15% [3,4]. Reportedly, 70–80% of nephrolithiasis is composed of calcium oxalate [5,6]. The primary treatment for kidney stones comprises of surgery, including extracorporeal shockwave lithotripsy, ureterorenoscopic lithotripsy, nephrolithotomy, and open surgery; these surgeries are complicated and expensive and do not affect the recurrence of stones. The 2012 estimates of the Urologic Diseases in America project reported that the cost is now up to $10 billion, rendering stone disease one of the most expensive urological conditions [7]. Reportedly, the recurrence rate of nephrolithiasis is approximately 50% in 10 years posttreatment [8]. Patients with nephrolithiasis bear a massive economic burden and face an additional risk of urinary tract obstruction, infection, kidney seeper, and renal insufficiency. Typically, proper management of diet and medications is required to prevent the first episodes of kidney stone formation or its secondary episodes. Dietary regulation includes decreasing animal protein, high sodium, and drinking plenty of water (1.9–2.8 L). While thiazide, citrate, or allopurinol helps to prevent kidney stone recurrence, the current treatment modalities do not effectively prevent urolithiasis. The sequence of events that triggers stone formation comprises of nucleation, growth, aggregation, and retention of crystals in the kidneys. Reportedly, controlling crystal-cell retentions is one of the best ways to treat urolithiasis [9]. Of note, NOX4 is a transmembrane transporter that transports electrons out of the serosa to produce superoxide and other reactive oxygen species (ROS). During the formation of kidney stones, calcium oxalate crystals activate NOX4 through the renin–angiotensin–aldosterone system, generating excessive ROS, which activates the p38 MAPK pathway, increases the expression of inflammatory factors osteopontin (OPN) and monocyte chemoattractant protein-1 (MCP-1), promotes the adhesion of calcium oxalate crystals and the formation of kidney stones [10,11,12,13].

With a history of 2000 years, Dai medicine originated in the Southeast Asia area, including southwestern Yunnan, eastern Myanmar, northern Thailand, central and northern Laos, and northwestern Vietnam, which is one of the areas with a high incidence of nephrolithiasis [4]. The treatment of kidney stones using Dai medicine involves plenty of herbs, of which *Aspidopterys obcordata* is effective. *A. obcordata* belongs to the Malpighiaceae Aspidoptery family, and the stems and branches are the medicinal part. *A. obcordata* has a long history in the folk application of the Dai people, used for the treatment of stones, acute and chronic nephritis, cystitis, and also used in the form of health tea for postpartum weight loss. The safety and effectiveness of the drug is reliable to some extent [14]. In our previous study, we observed the effects of different parts of *A. obcordata* on rats with calcium oxalate kidney stones. It was found that 95% ethanol extract of *A. obcordata* could reduce the volume of kidney stones and decrease serum creatinine and urea nitrogen levels in rats with kidney stones. The results of in vitro experiments also showed that the 95% ethanol site protected renal tubular epithelial cells from damage caused by calcium oxalate crystals, so the 95% ethanol site is considered to be an effective part of *A. obcordata* [15]. Later, some new polyoxypregnane glycosides were isolated from *A. obcordata*, which were named Obcordata [16]. Obcordata A (OA) is the same type of compound progesterone which can dilate the ureter and treat kidney stones [17]. However, the effect of OA on the formation of kidney stones is not clear. Hence, the human kidney 2 (HK-2) cell injury model was established using the leading component of kidney stones, calcium oxalate, to investigate the intervention effect of OA.

## 2. Results

### 2.1. Cytotoxicity of OA on HK-2 Cells

**Figure 1:**
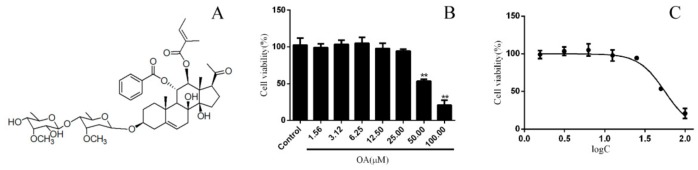
The cytotoxicity of Obcordata A (OA) on HK-2 cells. (**A**) The structural formula of OA. (**B**) The cytotoxicity to HK-2 cells at different concentrations of OA. Cells were incubated with different doses of OA for 24 h. Cell viability was measured using the MTT assay. OA has a significant inhibitory effect on the proliferation of HK-2 cells at concentrations above 50 μM. (**C**) The IC_50_ value of OA to HK-2 cells. The IC50 value was obtained by fitting cell viability with the drug concentration logarithm. n = 3 independent experiments. ** *P* < 0.05 versus control group.

Considering the potential cytotoxicity of OA in mammalian cells, HK-2 cells were treated with various doses of OA for 24 h, and cell viability was determined using the MTT assay. OA at 1.56–25.00 μM did not cause a significant change in cell viability (Figure 1B). Furthermore, the 50% inhibitory concentration (IC_50_) value was estimated as 55.89 μM (R^2^ = 0.9647). Then, nontoxic dosage (1–5 μM) was used for subsequent experiments (Figure 1C).

### 2.2. Protective Effects of OA on the Viability of HK-2 Cells Exposed to Calcium Oxalate Crystals

**Figure 2:**
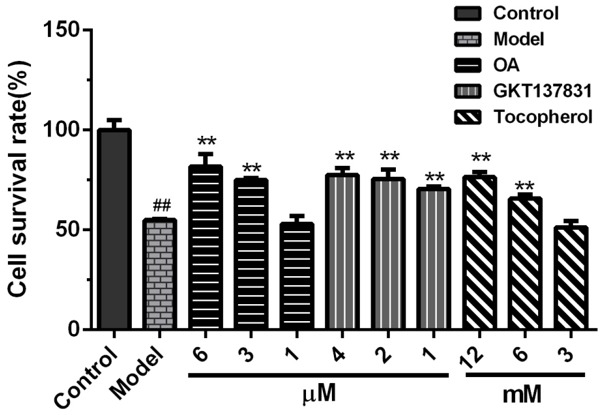
Protective effects of Obcordata A (OA) against calcium oxalate crystals induced HK-2 cell injury. OA and calcium oxalate crystals were simultaneously added to the medium, cultured for 24 h, and cell viability was measured using the MTT assay. All results are expressed as the mean SD of three independent experiments. Cell survival rate in the model group was significantly reduced, and OA, GKT137831, and tocopherol increased cell viability in a dose-dependent manner. ^##^ *P* < 0.01 versus control group; ** *P* < 0.01 versus model group.

The viability of HK-2 cells (54.88 ± 0.52%) declined compared with the control group (Figure 2). However, OA (5 and 2.5 μM) significantly increased cell viability (81.77 ± 6.24%, 75.09 ± 1.02%). The protective effect of OA was dose-dependent. Furthermore, NOX4 inhibitor, GKT13783, and antioxidant tocopherol markedly increased cell viability.

### 2.3. Inhibition of OA on the Intracellular Reactive Oxygen Species Production

**Figure 3:**
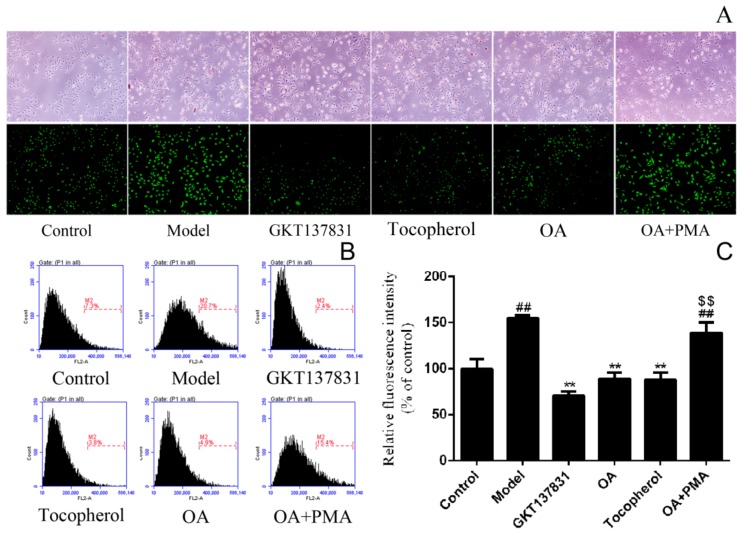
Obcordata A (OA) inhibits the intracellular reactive oxygen species (ROS) production. (**A**) The dichlorodihydrofluorescein diacetate (DCFH-DA) assay was performed to detect cellular ROS levels. Cell morphology and fluorescence intensity were observed and recorded using a microscope (original magnification, ×400). In the model group, the cells shrunk, the transmittance increased, and calcium oxalate crystals were observed on the cells and in the medium. In fluorescence mode, stronger fluorescence intensity means higher intracellular ROS levels. OA can reduce the fluorescence intensity of model cells, which can be eliminated by phorbol-12-myristate-13-acetate (PMA). (**B**) DCFH-DA staining in HK-2 cells was detected by flow cytometry. (**C**) Statistical results of relative fluorescence intensity. The mean fluorescence intensity of the cells in the model group was significantly increased, and OA, GKT137831, and tocopherol reduced the fluorescence intensity of the cells. When OA and PMA are added at the same time, the fluorescence intensity of the cells is not lowered. n = 3 independent experiments. ^##^ *P* < 0.01 versus control group; ** *P* < 0.01 versus model group; ^$$^ *P* < 0.01 versus OA group.

The relative fluorescence intensity of each group of cells is directly proportional to the level of intracellular reactive oxygen species (ROS). The ROS level of the model group increased significantly, reaching 154.7 ± 3.2% of the control group (Figure 3). In this study, OA, GKT13783, and tocopherol markedly decreased ROS levels compared with the model group. The trend of the fluorescent image corroborated the results of the flow cytometer. In addition, GKT13783 decreased ROS production by inhibiting the NOX4 expression. Tocopherol scavenges free radicals and, thus, decreases ROS levels at a higher concentration. In the presence of the NOX4 agonist phorbol-12-myristate-13-acetate (PMA), the effect of OA on the inhibition of ROS was diminished. Regarding concentration, OA is closer to GKT13783. Furthermore, DPPH scavenging experiments were conducted with tocopherol as a positive control to further determine its mechanism of action.

### 2.4. DPPH Radical Scavenging Activity of OA Was Low

**Figure 4:**
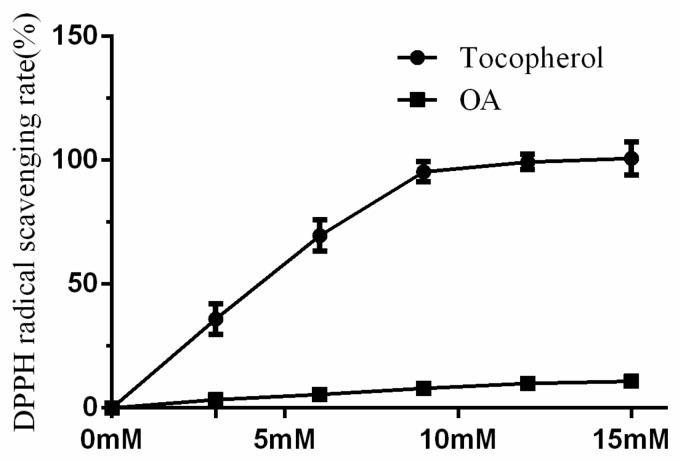
The DPPH radical scavenging test of Obcordata A (OA), investigates the dose–effect relationship of OA scavenging DPPH free radicals and judge its direct antioxidant activity. n = 3 independent experiments.

The DPPH radical scavenging activity of OA was significantly lower than tocopherol (3–15 mM). In addition, the DPPH radical scavenging rate of tocopherol (15 mM) was 100.8 ± 6.7%, while that of the same concentration OA was only 10.8 ± 1.3% (Figure 4).

### 2.5. OA Inhibits the Expression Level of Proteins in NOX4/ROS/p38 MAPK Signaling and Decreases ROS Levels

**Figure 5:**
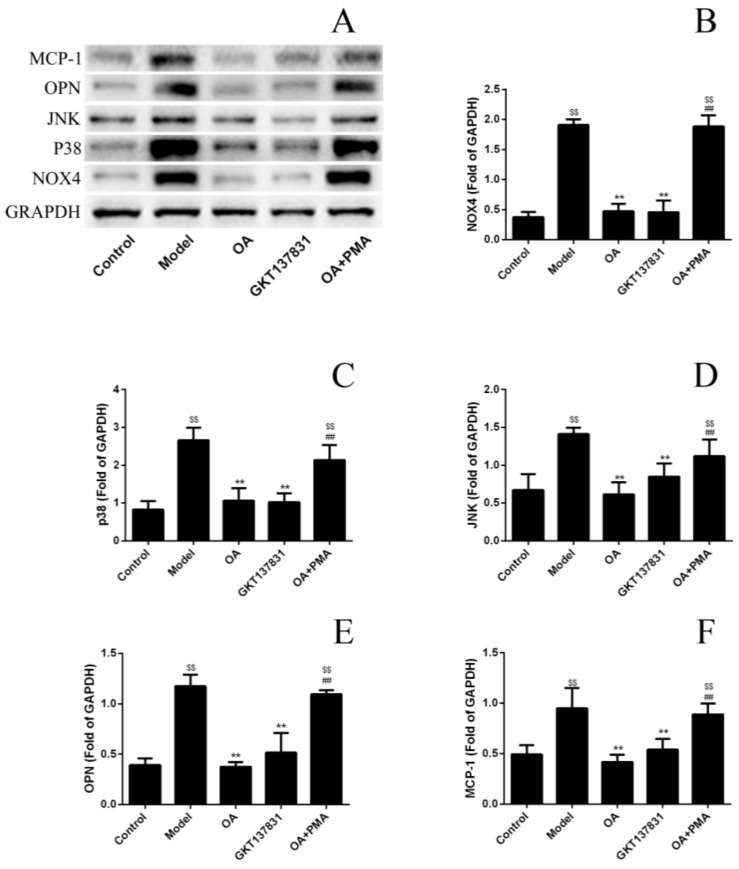
Effects of Obcordata A (OA) on the expression of the NOX4/ROS/p38 MAPK pathway and kidney stones–related proteins in HK-2 cells damaged by calcium oxalate crystals. (**A**) Immunoreactive protein bands representing glyceraldehyde 3-phosphate dehydrogenase (GAPDH), NOX4, p38, c-Jun N-terminal kinase (JNK), osteopontin (OPN), and monocyte chemoattractant protein-1 (MCP-1) relative expression. (**B**–**F**) Quantitative band intensity data statistical results of NOX4, P38, JNK, OPN, and MCP-1. OA can reduce the expression of NOX4 proteins and downgrade the NOX4/ROS/p38 MAPK pathway, thereby reducing the expression levels of downstream inflammatory factors MCP-1 and OPN. This mediation disappears after joining PMA. n = 3 independent experiments. ** *P* < 0.01 versus model group; ^$$^ *P* < 0.01 versus control group; ^##^ *P* < 0.01 versus OA group.

The NOX4 protein plays a vital role in kidney stone formation. The Western blot analysis was performed to examine the NOX4/ROS/p38 MAPK signaling proteins expression level and further confirm that OA could reduce ROS production by inhibiting the NOX4 protein expression. The expression of the NOX4 protein in the model group was markedly increased than that in the control group (Figure 5). NOX4 inhibitors GKT13783 and OA could inhibit the expression of NOX4 protein compared with the model group. The expression of NOX4 protein increased when the NOX4 agonist PMA and OA exited simultaneously (Figure 5B). Of note, proteins downstream of the NOX4/ROS/p38 MAPK pathway in each group exhibited the same trend. Correspondingly, OA could decrease ROS levels and the damage of calcium oxalate to HK-2 cells, compared with the model group; however, this effect was blocked by PMA.

## 3. Discussion

Globally, the incidence of kidney stones is increasing year by year, and one-quarter of patients are hospitalized for surgical treatment of renal colic or hematuria [18]. Kidney stones often relapse after treatment, which is expensive, thereby seriously affecting the patients’ quality of life [19]. Thus, preventing the occurrence and recurrence of kidney stones has been a major concern for patients and clinicians alike [20,21]. The formation of kidney stones comprises multiple processes, in which renal tubular epithelial structure destruction is the most critical step [22]. In healthy people, calcium oxalate in the urine crystallizes because of supersaturation but does not form stones. Evaluation of the growth rate of calcium oxalate crystals and the liquid flow rate of the renal tubules revealed that a single crystal could not increase to the extent of staying in the kidneys or occluding the renal tubules even when passing through the passage nephron and even at the maximum rate [23,24]. Thus, antagonizing renal tubular epithelial cell damage caused by various causes is one of the crucial means to prevent kidney stones. Growing evidence reveals that renal tubular epithelial cell damage during stone formation is primarily caused by ROS, which in the kidneys are mostly derived from NOX4 [10,25]. OA is a pregnane compound isolated from *A. obcordata*. This type of compound is generally reported as a cholinesterase inhibitor, which also has anti-proliferative effects and regulates drug metabolism [26,27,28,29]. In this study, OA markedly decreased intracellular ROS levels at 2.5–5 μM doses but did not exhibit significant DPPH scavenging activity. Subsequent Western blotting experiments established that OA downregulated the NOX4/ROS/p38 MAPK pathway, inhibited ROS production, and alleviated HK-2 cell damage. To the best of our knowledge, this is the first demonstration that progesterone compounds can inhibit NOX4 expression. OA inhibits NOX4 expression and avoids adhesion of calcium oxalate crystals, possibly to be used for the prevention of kidney stones.

However, there are still many limitations in this study. Calcium oxalate–induced cell damage includes various processes of apoptosis, autophagy, inflammatory, and necrosis, which is mainly affected by the size of the model with calcium oxalate crystals [30,31]. In our study, only the inflammatory response was focused on. Evaluation of OA antioxidant activity is not comprehensive. In future studies, the effect of OA in preventing kidney stones will be examined in animal models. Additionally, we will evaluate efficacy by renal pathological changes and number of stones, and investigate the mechanism of drug intervention in various injury processes of renal tubular epithelial cells. It is hoped that the research can provide a basis for the prevention of kidney stones in *Aspidopterys obcordata* vines. Patients with kidney stones should be taken regularly after treatment to avoid recurrence.

## 4. Materials and Methods

### 4.1. Antibodies and Reagents

The antibodies against NOX4, P38, JNK, OPN, MCP-1, and glyceraldehyde 3-phosphate dehydrogenase (GAPDH) were obtained from OriGene Technologies (Rockville, MD, USA). Goat anti-rabbit and goat anti-mouse immunoglobulin G, protein extraction reagent, were purchased from CW Biotech (Beijing, China). GKT137831was purchased from Selleck Chemicals (Selleck Chemicals, Houston, TX, USA), while phorbol-12-myristate-13-acetate (PMA) and dichlorodihydrofluorescein diacetate (DCFH-DA) were acquired from Beyotime Biotechnology (Beyotime Institute of Biotechnology, Shanghai, China). Furthermore, Dulbecco’s modified Eagle’s medium (DMEM), fetal bovine serum (FBS), and penicillin–streptomycin solution were obtained from HyClone (Thermo Fisher Scientific, Waltham, MA, USA). Dimethyl sulfoxide (DMSO) was provided by MP Biomedicals, LLC (MP Biomedicals, LLC, Santa Ana, CA, USA). OA was donated by Dr. Meigeng Hu from the Institute of Medicinal Plant Development, and the purity was 95%. In cell experiments, OA was dissolved in DMSO to prepare a 20 mmol solution, which was diluted to the desired concentration using DMEM medium. In DPPH radical scavenging assay, OA was dissolved in ethanol.

### 4.2. Cell Culture

HK-2 cells provided by the Procell Life Science & Technology Co., Ltd. (Wuhan, China) were cultured in DMEM supplemented with 10% FBS, 1% penicillin–streptomycin solution, and placed in a CO_2_ incubator at 37 °C with 5% CO_2_.

### 4.3. Cytotoxicity Assay of OA

HK-2 cells were seeded (2 × 10^4^ cells/mL) in 96-well microplates and treated with OA in different concentrations of 6.25, 12.5, 25, 50, and 100 μM for 24 h. Then, the cytotoxicity of OA was assessed with the MTT assay, as described previously [32]. Furthermore, OD values were read at 570 nm by an automatic multifunctional microplate reader (Model i3; Molecular Devices, San Jose, CA, USA).

### 4.4. Calcium Oxalate Crystals Exposure and Treatment

Calcium oxalate monohydrate (COM) crystals were prepared as described previously with little modification [33]. Briefly, 10 mmol/mL sodium oxalate was mixed with the same volume 10 mmol/mL CaCl_2_ and allowed to stand at room temperature for 10 min. We collected COM crystals by centrifugation at 1000 × *g* for 10 min and dried in an oven at 60 °C. Then, HK-2 cells in 96-well microplates were exposed to UV-sterilized COM crystals at a density of 28 μg·cm^2^ [11]; 1, 2.5, and 5 μM OA were used in this step for the dose-dependent study. Of note, cells without any treatment served as the control. Different concentrations of GKT137831 and tocopherol were used as a positive control [34]. The cell viability was detected after 24 h using the MTT assay.

### 4.5. Intracellular Reactive Oxygen Species Production by DCFH-DA Assay, Fluorescence Microscopy, and Flow Cytometry

HK-2 cells were seeded in 60-mm Petri dishes and exposed to COM crystals alone or COM crystals and drugs together. The doses of OA, GKT137831, PMA, and tocopherol were 2.5, 2.5, 1 μM, and 6 mM, respectively [35,36]. After 24-h incubation, cells were incubated in serum-free medium with 10 μmol/L DCFH-DA probe for 20 min. The probe was removed by changing the medium three times. The cell morphology and fluorescence intensity at 488 nm were observed and recorded using a Nikon microscope (Nikon Instruments Inc., Melville, NY, USA).

For flow cytometry, we harvested cells by trypsinization. The DCFH-DA probe was loaded per the manufacturer’s instructions. Briefly, cells were suspended with DMEM containing 10 μmol/L DCFH-DA probe, incubated at 37 °C for 30 min, and gently inverted every 5 min. Then, cells were collected by centrifugation at 70 × *g* and washed with serum-free medium three times. Next, the fluorescence of cells was detected using a BD Accuri C6 flow cytometer (Becton, Dickinson and Company, Franklin Lakes, NJ, USA). The experimental results were expressed as the ratio of the average fluorescence intensity of each group to the blank control group—relative fluorescence intensity. The average fluorescence intensity was analyzed using Image-Pro Plus 6.0 software (Media Cybernetics, Rockville, MD, USA).

### 4.6. DPPH Radical Scavenging Test

The DPPH radical scavenging capacity of OA was determined as described previously with modification [37]. Briefly, 180 μL of DPPH anhydrous ethanol solution (10 μg/mL) was added to each well of a 96-well plate. Five set concentrations of OA and tocopherol were 3, 6, 9, 12, and 15 μM; three replicates per concentration, and the reaction system was 200 μL. Then, 20 μL of ethanol was added to control wells, and an equal volume of ethanol was used as background. After 30 min in the dark, the absorbance was measured at 515 nm. After deducting the background, the percentage of decrease in the absorbance value compared with the control group was considered the DPPH radical scavenging rate.

### 4.7. Western Blots Assay

The expression of NOX4, P38, JNK, MCP-1, and OPN proteins were evaluated by Western blot analysis. HK-2 cells in 60-mm Petri dishes were established as the calcium oxalate damage model based on the method described above. Simultaneously, OA, GKT137831, or OA, along with PMA, were added to the medium. After 24-h incubation, cells were harvested and washed with ice-cold PBS, incubated on ice for 20 min in mammalian protein extraction reagent containing 1% protease inhibitor cocktail (CW Biotech, Beijing, China). Then, the supernatant was collected after centrifugation at 12,000 × *g* for 15 min. The protein concentration in the supernatant was measured by the BCA Protein Quantification Kit (CW Biotech) and diluted to a uniform concentration by adding the protein extraction reagent. The loading buffer (CW Biotech) was added to the protein sample at a ratio of 1:4, and added to a boiling water bath for 5 min. Next, equal amounts of protein samples (10 μL) were resolved using sodium dodecyl sulfate–polyacrylamide gel electrophoresis (SDS–PAGE; 8–12% acrylamide gels) and transferred to polyvinylidene difluoride (PVDF) membrane (Millipore, Waltham, MA, USA). Then, membranes were blocked in medium containing 5% non-fat milk tris-buffered saline with Tween 20 (TBST) solution at room temperature for 2 h and, then, incubated with primary antibodies at 4 °C. All membranes were washed three times with TBST solution and incubated with secondary antibodies at room temperature for 2 h. We visualized protein bands by enhanced chemiluminescence (CW Biotech) and analyzed by densitometry using ImageQuant TL software (GE Healthcare, Uppsala, Sweden).

### 4.8. Statistical Analysis

In this study, all quantitative data were presented as mean ± SEM from three independent experiments. The mean differences between multiple comparisons among several groups were analyzed by one-way ANOVA with Tukey’s post-hoc test. We set a statistically significant threshold at *p* < 0.05. Furthermore, the IC_50_ value was obtained using log (inhibitor) versus normalized response—variable slope in nonlinear regression (curve fit).

## 5. Conclusions

OA inhibits NOX expression, reduces ROS production, regulates NOX4/ROS/p38 MAPK pathway, reduces inflammatory factor levels, and protects renal tubular epithelial cells from calcium oxalate crystal damage and adhesion.

## References

[1] Wang W., Fan J., Huang G., Li J., Zhu X., Tian Y., Su L. (2017). Prevalence of kidney stones in mainland China: A systematic review. Sci. Rep..

[2] Januchowski R., Dabecco R., Verdone C. (2014). Nephrolithiasis. Osteopath. Fam. Physician.

[3] Sorokin I., Mamoulakis C., Miyazawa K., Rodgers A., Talati J., Lotan Y. (2017). Epidemiology of stone disease across the world. World J. Urol..

[4] Hernandez J.D., Ellison J., Lendvay T.S., Hernandez M.J.D., Ellison M.J.S., Lendvay M.T.S. (2015). Current Trends, Evaluation, and Management of Pediatric Nephrolithiasis. JAMA Pediatr..

[5] Jiang Z., Asplin J.R., Evan A.P., Rajendran V.M., Velazquez H., Nottoli T.P., Binder H.J., Aronson P.S. (2006). Calcium oxalate urolithiasis in mice lacking anion transporter Slc26a6. Nat. Genet..

[6] Indridason O.S., Birgisson S., Edvardsson V.O., Sigvaldason H., Sigfusson N., Palsson R. (2006). Epidemiology of kidney stones in Iceland A population-based study. Scand. J. Urol. Nephrol..

[7] Lotan Y., Cadeddu J.A., Pearle M.S. (2005). International comparison of cost effectiveness of medical management strategies for nephrolithiasis. Urol. Res..

[8] Bushinsky D., Michalenka A., Strutz K., Donahue S., Asplin J. (2008). Effect of bolus and divided feeding on urine ions and supersaturation in genetic hypercalciuric stone-forming rats. Kidney Int..

[9] Dhayat N.A., Faller N., Bonny O., Mohebbi N., Ritter A., Pellegrini L., Bedino G., Schönholzer C., Venzin R.M., Hüsler C. (2018). Efficacy of standard and low dose hydrochlorothiazide in the recurrence prevention of calcium nephrolithiasis (NOSTONE trial): Protocol for a randomized double-blind placebo-controlled trial. BMC Nephrol..

[10] Khan S.R. (2012). Is oxidative stress, a link between nephrolithiasis and obesity, hypertension, diabetes, chronic kidney disease, metabolic syndrome?. Urol. Res..

[11] Koul H.K., Menon M., Chaturvedi L.S., Sekhon A., Bhandari A., Huang M., Koul S. (2002). COM Crystals Activate the p38 Mitogen-activated Protein Kinase Signal Transduction Pathway in Renal Epithelial Cells. J. Boil. Chem..

[12] Zuo J., Khan A., Glenton P.A., Khan S.R. (2011). Effect of NADPH oxidase inhibition on the expression of kidney injury molecule and calcium oxalate crystal deposition in hydroxy-l-proline-induced hyperoxaluria in the male Sprague–Dawley rats. Nephrol. Dial. Transplant..

[13] Qin B., Wang Q., Lu Y., Li C., Hu H., Zhang J., Wang Y., Zhu J., Zhu Y., Xun Y. (2018). Losartan Ameliorates Calcium Oxalate-Induced Elevation of Stone-Related Proteins in Renal Tubular Cells by Inhibiting NADPH Oxidase and Oxidative Stress. Oxid. Med. Cell. Longev..

[14] Yihang L., Guang L., Meifang S., Xuelan L., Xia Z., Juan L., Xi C. (2016). Acute toxicity study of *Aspidopterys obcordata* aqueous extract in Sprague-Dawley rats. J. Tradit. Chin. Med..

[15] Song M.-F., Li Y.-H., Zhang Z.-L., Lü Y.-N., Li X.-L., Li G. (2015). Inhibiting effect of *Aspidopterys obcordata* Hemsl on renal calculus. Chin. J. New Drugs.

[16] Hu M., Li Y., Sun Z., Huo X., Zhu N., Sun Z., Liu Y., Wu H., Xu X., Ma G. (2018). New polyoxypregnane glycosides from *Aspidopterys obcordata* vines with antitumor activity. Fitoterapia.

[17] Healy K., Ogan K. (2005). Nonsurgical Management of Urolithiasis: An Overview of Expulsive Therapy. J. Endourol..

[18] Alelign T., Petros B. (2018). Kidney Stone Disease: An Update on Current Concepts. Adv. Urol..

[19] Leveridge M., D’Arcy F.T., O’Kane D., Ischia J.J., Webb D.R., Bolton D.M., Lawrentschuk N. (2016). Renal colic: Current protocols for emergency presentations. Eur. J. Emerg. Med..

[20] Wang L., Feng C., Ding G., Lin X., Gao P., Jiang H., Xu J., Ding Q., Wu Z. (2017). Association Study of Reported Significant Loci at 5q35.3, 7p14.3, 13q14.1 and 16p12.3 with Urolithiasis in Chinese Han Ethnicity. Sci. Rep..

[21] Rimer J.D., An Z., Zhu Z., Lee M.H., Goldfarb D.S., Wesson J.A., Ward M.D. (2010). Crystal Growth Inhibitors for the Prevention of L-Cystine Kidney Stones through Molecular Design. Science.

[22] Khan S.R. (2014). Reactive oxygen species, inflammation and calcium oxalate nephrolithiasis. Transl. Androl. Urol..

[23] Tsujihata M. (2008). Mechanism of calcium oxalate renal stone formation and renal tubular cell injury. Int. J. Urol..

[24] Dörrenhaus A., Müller J.I., Golka K., Jedrusik P., Schulze H., Föllmann W. (2000). Cultures of exfoliated epithelial cells from different locations of the human urinary tract and the renal tubular system. Arch. Toxicol..

[25] Lee H.-J., Jeong S.-J., Park M.N., Linnes M., Han H.J., Kim J.H., Lieske J.C., Kim S.-H. (2012). Gallotannin Suppresses Calcium Oxalate Crystal Binding and Oxalate-Induced Oxidative Stress in Renal Epithelial Cells. Boil. Pharm..

[26] Devkota K.P., Lenta B.N., Choudhary M.I., Naz Q., Fekam F.B., Rosenthal P.J., Sewald N. (2007). Cholinesterase Inhibiting and Antiplasmodial Steroidal Alkaloids from Sarcococca hookeriana. Chem. Pharm..

[27] Zhao H.-Y., Shao C.-L., Li Z.-Y., Han L., Cao F., Wang C.-Y. (2013). Bioactive Pregnane Steroids from a South China Sea Gorgonian Carijoa sp.. Molecules.

[28] Orans J., Teotico D.G., Redinbo M.R. (2005). The Nuclear Xenobiotic Receptor Pregnane X Receptor: Recent Insights and New Challenges. Mol. Endocrinol..

[29] Hussain H., Raees M.A., Rehman N.U., Al-Rawahi A., Csuk R., Khan H.Y., Abbas G., Al-Broumi M.A., Green I.R., Elyassi A. (2015). Nizwaside: A new anticancer pregnane glycoside from the sap of Desmidorchis flava. Arch. Pharmacal.

[30] Sun X.-Y., Ouyang J.-M. (2015). New view in cell death mode: Effect of crystal size in renal epithelial cells. Cell Death.

[31] Grohm J., Kim S.-W., Mamrak U., Tobaben S., Cassidy-Stone A., Nunnari J., Plesnila N., Culmsee C. (2012). Inhibition of Drp1 provides neuroprotection in vitro and in vivo. Cell Death Differ..

[32] Li G., Xing X., Luo Y., Deng X., Lu S., Tang S., Sun G., Sun X. (2018). Notoginsenoside R1 prevents H9c2 cardiomyocytes apoptosis against hypoxia/reoxygenation via the ERs/PI3K/Akt pathway. RSC Adv..

[33] Wiessner J.H., Hung L.Y., Mandel N.S. (2003). Crystal attachment to injured renal collecting duct cells: Influence of urine proteins and pH. Kidney Int..

[34] Jeong B.Y., Lee H.Y., Park C.G., Kang J., Yu S.L., Choi D., Han S.Y., Park M.H., Cho S., Lee S.Y. (2018). Oxidative stress caused by activation of NADPH oxidase 4 promotes contrast-induced acute kidney injury. PLoS ONE.

[35] Von Lohneysen K., Noack D., Wood M.R., Friedman J.S., Knaus U.G. (2010). Structural Insights into Nox4 and Nox2: Motifs Involved in Function and Cellular Localization. Mol. Cell. Biol..

[36] Serrander L., Cartier L., Bedard K., Bánfi B., Lardy B., Plastre O., Sienkiewicz A., Forró L., Schlegel W., Krause K.-H. (2007). NOX4 activity is determined by mRNA levels and reveals a unique pattern of ROS generation. Biochem. J..

[37] Villaño D., Fernández-Pachón M., Moyá M.L., Troncoso A.M., Garcia-Parrilla M.C. (2007). Radical scavenging ability of polyphenolic compounds towards DPPH free radical. Talanta.

